# Protease-activated receptor 4 deficiency increases mortality, intracranial bleeding, and blood-brain barrier impairment following traumatic brain injury in mice

**DOI:** 10.1016/j.rpth.2025.103238

**Published:** 2025-10-30

**Authors:** Leonie S. Link, Christina Gölz, Regina Hummel, Katharina Ritter, Sabine Reyda, Wolfram Ruf, Michael K.E. Schäfer, Eva-Verena Griemert

**Affiliations:** 1 Department of Anesthesiology, University Medical Center of the Johannes Gutenberg University Mainz, Mainz, Germany; 2 Center for Thrombosis and Hemostasis, University Medical Center of the Johannes Gutenberg University Mainz, Mainz, Germany

**Keywords:** Blood-brain barrier, coagulopathy, intracerebral hemorrhage, protease-activated receptor, traumatic brain injury

## Abstract

**Background:**

Protease-activated receptors (PARs), comprising PAR1 to PAR4, play a critical role in hemostasis and are considered potential therapeutic targets. Clinical trials have evaluated the safety and dosage of PAR4 antagonists, and a pathogenic role of PAR4 has been demonstrated in animal models of coagulopathies, including hemorrhagic brain injury. However, there is a lack of studies regarding the acute effects of PAR4 deficiency (PAR4^-/-^) following experimental traumatic brain injury (TBI).

**Objectives:**

To determine the role of PAR4 in cerebral hemostasis after TBI.

**Methods:**

Adult male PAR4^-/-^ and genetically background-matched wild-type C57BL/6 mice (BL6; *N* = 22 each) were subjected to the controlled cortical impact model of TBI. We analyzed physiological and neurological parameters as well as (immuno-)histology and gene expression.

**Results:**

PAR4^-/-^ mice exhibited increased mortality and body weight loss within 24 hours after TBI compared with BL6 mice. TBI-induced neurological deficits were similar between PAR4^-/-^ and BL6 mice. Notably, PAR4^-/-^ mice exhibited subdural hematoma, increased intracerebral hemorrhage, and blood-brain barrier leakage compared with BL6 mice. Severely impaired hemostasis was not associated with significant changes in brain lesion size or in the inflammatory activation of microglia and astrocytes in surviving mice.

**Conclusion:**

Our results demonstrate a crucial role for PAR4 in cerebral hemostasis following experimental TBI in mice and suggest that particular caution is warranted in the therapeutic management of PAR4-targeted treatment of coagulopathies.

## Essentials

• We determined the effect of PAR4^-/-^ on cerebral hemostasis after TBI.
• We analyzed neurological, histological, and genetic parameters after controlled cortical impact.
• PAR4^-/-^ mice exhibited higher mortality and impaired cerebral hemostasis and blood-brain barrier.
• Caution may be required in the management of PAR4-targeted treatment of coagulopathies after TBI.

## 1 Introduction

The maintenance of a precise balance between blood coagulation and fibrinolysis is crucial to prevent both hypercoagulability and increased bleeding risk following traumatic brain injury (TBI) [1,2]. TBI-induced coagulopathy occurs early and manifests as disseminated intracranial hemorrhage, delayed intracranial or intracerebral hematoma, and systemic bleeding and is strongly correlated with poor clinical outcomes [3]. The excessive release of tissue factor (TF), hyperactivity of platelets, and an increase in membrane microparticles and extracellular vesicles have been associated with coagulopathy after brain injury [[4], [5], [6]]. Findings from experimental TBI models, such as controlled cortical impact (CCI) and fluid percussion injury, further indicate that TBI rapidly activates the TF pathway, resulting in thrombin generation, platelet aggregation, and fibrin deposition, while also increasing fibrinolysis [[7], [8], [9], [10]]. These and other studies have also examined the potential link between injury-induced coagulopathy and inflammation, as immune cells and coagulation factors exhibit reciprocal interactions [11,12]. Hence, the selective modulation of specific coagulation factors or fibrinolytic enzymes may potentially confer neuroprotective and anti-inflammatory effects during the acute phase of TBI.

Among the prime candidates are protease-activated receptors (PARs). PARs belong to the 7 transmembrane-spanning G protein-coupled receptor superfamily, and comprise PAR1 to PAR4 (encoded by the genes F2R, F2RL1, F2RL2, and F2RL3, respectively). PARs are cleaved by coagulation proteases released from monocytes/macrophages, thereby connecting hemostasis with innate immune responses [[13], [14], [15], [16]]. The pharmacological inhibition of PAR1 is used in patients who suffer from myocardial infarction or peripheral arterial disease to prevent cardiovascular death or cerebral ischemia, while, however, increasing the risk of bleeding, including intracranial hemorrhage [17]. More recently, selective PAR4 inhibition has been proposed as a particularly promising approach due to its efficacy and safety considerations in the context of acute thrombosis, as well as its potential anti-inflammatory effects [18]. Accordingly, recent clinical trials with novel PAR4 antagonists, ie, BMS-986141 and BMS-986120, found them to be safe and well-tolerated over a wide dose range [19,20].

In contrast to human platelets that are activated by PAR1 and PAR4, platelets from PAR4-deficient (PAR4^-/-^) mice are unresponsive to thrombin due to a complete lack of thrombin signaling [21]. This finding was extended using conditional PAR4^-/-^ in megakaryocytes/platelets following vein laser injury [22] or constitutive PAR4^-/-^ following ferric chloride–induced arterial thrombosis [23]. In the context of acute brain injuries, PAR4^-/-^ was reported to inhibit both platelet activation and microvascular inflammation, resulting in reduced infarct volume 23 hours following transient middle cerebral artery occlusion (tMCAO) in mice [24]. Pharmacologic PAR4 inhibition also resulted in robust neuroprotective effects at 24 and 72 hours following tMCAO, and the authors suggested that inhibition of neuronal PAR4 confers postischemic neuroprotection [25]. However, the study design did not exclude a platelet-dependent effect. Indeed, recent investigations of a PAR4 variant demonstrated a platelet-dependent role of PAR4 in stroke outcome [26]. Following experimental TBI, PAR4 mRNA was induced in brain regions affected by apoptosis and neuronal cell loss [27], and the overexpression of PAR4 was associated with exacerbated neuronal cell death [28]. Accordingly, administration of the PAR4 inhibitor BMS-986120, which has a weak affinity for mouse platelets, may attenuate structural brain damage and neuronal cell death following TBI in mice [29].

The accumulated evidence identified PAR4 as a deleterious but druggable factor at the interface of hemostasis, neuronal survival, and inflammation following brain injury. To date, no studies have investigated PAR4^-/-^ mice in experimental TBI. However, such studies may provide valuable insights, as a PAR4^-/-^ model may effectively simulate a scenario of maximal pharmacological inhibition of PAR4. To this end, PAR4^-/-^ and wild-type (BL6) mice were subjected to the CCI model of TBI and examined for neurological deficits and key neuropathological features, including structural brain damage, blood-brain barrier (BBB) impairment, and the expression of inflammatory markers.

## 2 Methods

### 2.1 Animals

Animal care and experimental procedures complied with the institutional guidelines of the Johannes Gutenberg University Mainz, Germany, and were approved by the Animal Care and Ethics Committee of the Landesuntersuchungsamt Rheinland-Pfalz (protocol number: G 16-1-079). Experiments were performed according to the ARRIVE (Animal Research: Reporting of *In Vivo* Experiments) guidelines [30]. Adult male PAR4^-/-^ (B6[Cg]-F2rl3tm1.1 Cgh/Tarc) and background-matched adult male BL6 (C57BL/6) mice (∼25 g, 15-16 weeks old, *N* = 22 each) were maintained and bred in the institutional animal facilities (University Medical Center Mainz, Germany). The mice were housed in a controlled environment (12-hour dark/light cycle, 23 ± 1 °C, and 55% ± 5% relative humidity; values are presented as mean ± SD) with food and water *ad libitum*. The observation period ended after 24 hours.

Experimenters performing the CCI procedure were blind to the experimental groups. All postinterventional analyses were performed in a blinded and unbiased fashion.

### 2.2 Experimental TBI, physiological parameters, and neurological assessment

The CCI method yields consistent intralaboratory outcomes due to precise control of the depth of cortical penetration, dwell time, and the velocity of impact. The CCI model was used to induce experimental TBI, essentially as described [31]. Briefly, mice were anesthetized with isoflurane (4% v/v for 60 seconds of induction and 2% v/v maintenance), and an electromagnetically controlled stereotaxic impactor (Impact One, Leica BioSystems) induced standardized TBI (3 mm impactor tip diameter, 6 m/s velocity, 200 ms duration, and 1.5 mm impact depth). The craniotomy and the skin were carefully closed, and the mice were subsequently transferred to their cages, then placed in a heated incubator (Dräger) with controlled air temperature (35 °C) and humidity (35%) for 1.5 hours.

**Figure 1:**
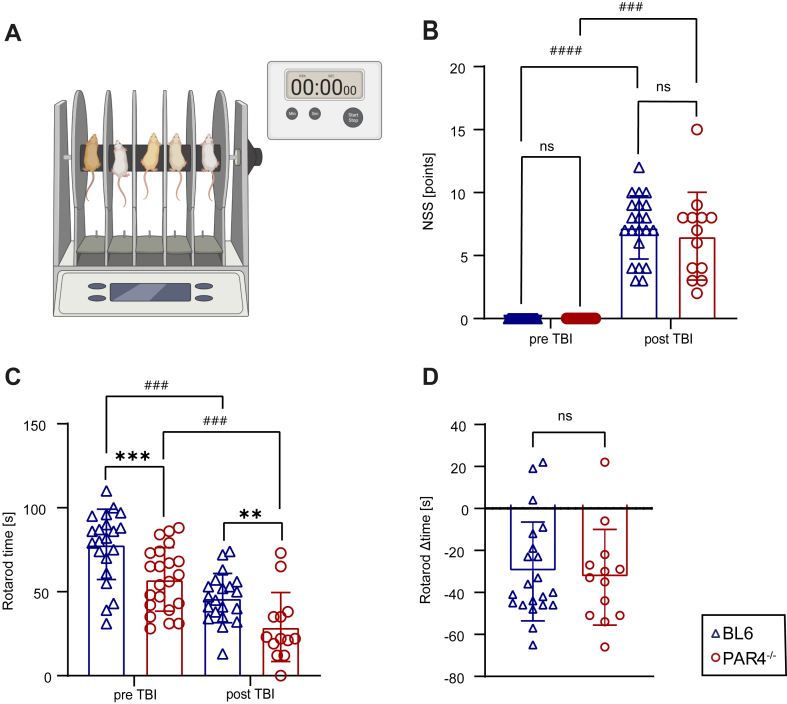
PAR4 deficiency (PAR4^-/-^) influences motoric but not neurological function in mice. (A) Schematic illustration of the Rotarod device. (B) Results of the Neurological Severity Score (NSS) before and after traumatic brain injury (TBI). Four outliers were identified by the ROUT test and excluded. Wild-type C57BL/6 (BL6) mice *n* = 21 pre TBI (1 oulier) and *n* = 21 post TBI (1 dead) ; PAR4^-/-^ mice: *n* = 19 pre TBI (3 outliers) and *n* = 13 post TBI (9 dead); analysis by the Mann–Whitney U-test, Student’s unpaired *t*-test, or Wilcoxon signed-rank test. (C) PAR4^-/-^ mice showed limited motoric skills before and after TBI in comparison with BL6 mice. One outlier was identified by the ROUT test and excluded. BL6: *n* = 22 pre TBI and *n* = 20 post TBI (1 outlier, 1 dead); PAR4^-/-^: *n* = 22 pre TBI and *n* = 13 post TBI (9 dead); analysis by the Mann–Whitney U-test, Student’s unpaired *t*-test, or Wilcoxon signed-rank test. (D) There was no significant difference in the Rotarod scores before and after TBI between PAR4^-/-^ and BL6 mice. BL6: *n* = 21; PAR4^-/-^: *n* = 13; analysis by the Mann–Whitney U-test. Values from individual animals and the mean ± SD are shown. ∗Indicates PAR4^-/-^ vs BL6; ∗*P* < .05; ∗∗*P* < .01; ∗∗∗*P* < .001; ∗∗∗∗*P* < .0001. #Indicates PAR4^-/-^/BL6 pre vs PAR4^-/-^/BL6 post; #*P* < .05; ##*P* < .01; ###*P* < .001; ####*P* < .0001; ns, not significant. Created with BioRender.com.

Physiological parameters, including pericranial and rectal body temperature, were analyzed prior to and during the operative procedure and maintained. The body weight was measured before and 24 hours after the experiment. Mice were assessed preoperatively and at the end of the observation period using the modified Neurological Severity Score (NSS), which consists of different tasks evaluating motor function, reflexes, alertness, balance, and general behavior [32]. In this study, a modified 16-point scale was used, ranging from 0 (no neurological impairment) to 16 (severe neurological dysfunction). At the previously mentioned time points, motor coordination and balance were likewise monitored using the Rotarod (RR) test (Panlab, Harvard Apparatus; Figure 1A). Mice were placed on an accelerating rotating cylinder, and the latency to fall was recorded as described [33].

### 2.3 Histological evaluation of brain damage

Mice were euthanized 24 hours after CCI under deep anesthesia (4% v/v isoflurane for 90 seconds). Brains were carefully removed, immediately frozen with powdered dry ice, and stored at −20 °C. For further histological examination, coronal cryosections were cut with a cryostat (HM 560 CryoStar, Thermo Fisher Scientific). In sum, 16 serial cryosections (12 μm thickness) per brain were collected at 500-μm intervals and placed on microscope slides (SuperFrost Plus, Menzel-Gläser) as outlined before [31,34]. Cryosectioning was started at bregma +3.14 mm according to the Mouse Brain Library atlas [35]. Tissue sampling for real-time quantitative polymerase chain reaction was carried out as described before [36]. To measure the extent of the brain lesion, slides were stained with cresyl violet (Merck), and the brain lesion volume was determined as previously described [36,37]. Data were expressed as the absolute contusion volume. The thickness of the dentate gyrus granule cell layer (GCL) is a reliable neuropathological indicator of brain damage distant from the direct lesion site in our TBI model [31]. The thickness of the GCL of the ipsilesional hippocampus was measured at bregma levels −2.86 mm and −3.36 mm at corresponding sites in the contralesional hemisphere. Mean values were calculated from measurements of 3 approximately equidistant locations (from the crest to the lateral tip), and the ipsilesional hemisphere was set at a ratio to the contralesional hemisphere [38].

Hematoxylin and eosin staining of 4 cryosections from bregma level +0.14 mm to −1.36 mm was used to assess hematoma formation after TBI, as described [36].

### 2.4 Immunostaining of immunoglobulin G

To assess BBB impairment, slides of bregma levels −1.86 mm and −2.36 mm were immunostained using anti-mouse immunoglobulin G (IgG) [39]. Briefly, cryosections were air-dried, fixed, and stained with horseradish peroxidase-conjugated goat anti-mouse IgG antibody (1:500; Santa Cruz Biotechnology, RRID: AB_631737). Images were quantified using a bright-field microscope (Stemi 305, Zeiss) and Zen software (Zeiss, RRID:SCR_013672). The brain regions with solid, dark IgG immunostaining, representing IgG extravasation (clearly distinguishable from regions without extravasation, which showed brown-colored blood vessels), were quantified as a percentage of the total brain volume.

### 2.5 Immunostaining of CD41 and Claudin-5

Immunostaining was performed essentially as described [7,37]. Briefly, coronal brain cryosections (12 μm) were air-dried, fixed in 4% paraformaldehyde in phosphate-buffered saline (PBS), and incubated in blocking solution (5% normal goat serum, 0.5% bovine serum, and 0.1% Triton X-100 (Sigma-Aldrich) in PBS) for 1 hour at room temperature. Primary antibodies specific to CD41 (host species: rat, catalog [Cat] number LS-C44479, Biozol) or Claudin-5 (host species: rabbit, Cat number 34-1600, Invitrogen) were diluted 1:100 in blocking solution and incubated at 4 °C overnight. The following day, sections were washed in PBS, incubated with secondary antibodies (goat anti-rat IgG [H + L] cross-adsorbed secondary antibody, Alexa Fluor 568, Thermo Fisher Scientific, Cat number A-11006, dilution: 1:1000; goat anti-rabbit IgG [H + L] cross-adsorbed secondary antibody, Alexa Fluor 488, Thermo Fisher Scientific, Cat number A-11011, dilution: 1:1000) in blocking solution for 2 hours at room temperature, counterstained with 4′,6-diamidino-2-phenylindol (Sigma-Aldrich), and mounted in ImmunoMount (Thermo Fisher Scientific). Images of the perilesional lateral cortex were captured from 2 brain sections of each animal (bregma −1.86 mm to −2.86 mm) using fluorescence microscopy (BZ-X800, Keyence). ImageJ (National Institutes of Health) was used for quantitative analysis of immunofluorescence signals, and mean values from 2 sections per animal (BL6: *n* = 10; PAR4^-/-^: *n* = 6) were expressed as anti-CD41 percentage of anti–Claudin-5 immunofluorescent area or percentage of anti–Claudin-5 nonimmunofluorescent area as a proxy for the presence of intravasal and extravasal platelets.

### 2.6 Gene expression analysis

Brain tissue was collected during serial cryosectioning and processed for gene expression analysis as described [36]. Briefly, RNA extraction from brain tissue and reverse transcription were performed using the RNeasy Kit and Quant iScript Reverse Transcription Kit (Qiagen), respectively. The produced complementary DNA was amplified in equal quantities by real-time quantitative polymerase chain reaction using a LightCycler 480 (F. Hoffmann-La Roche AG). The analysis was done by normalizing all values to the housekeeping gene Ppia, and absolute quantification was performed utilizing a target-specific standard curve of mRNA copies and the LightCycler software (F. Hoffmann-La Roche AG, RRID:SCR_012155) [40]. Sequences of applied oligonucleotide primer pairs, product lengths, and corresponding kits are listed in the Table.

**Table:** Primers used in real-time quantitative polymerase chain reaction.

Gene name, (amplicon size [bp], annealing temperature)	Oligonucleotide sequences 5′-3′	Kits (inclusive company)	GenBank number
*Cd68* (113 bp, 58 °C)	Fw: CCCACCTGTCTCTCTCATTTC Rev: CACATTGTATTCCACCGCC	PrimaQuant CYBR SL9902B (Steinbrenner Laborsysteme)	NM_001291058.1
*Fcgr1* (211 bp, 58 °C)	Fw: CCACAATGATTGGCTGCTACT Rev: CGTGCCTGAGCAGTGGTA	Absolute Blue qPCR SybrgreenMix AB-4166 (Thermo Fisher Scientific)	NM_010186.5
*Gfap* (120 bp, 58 °C)	Fw: CGGAGACGCATCACCTCTG Rev: TGGAGGAGTCATTCGAGACAA	Absolute Blue qPCR SybrgreenMix AB-4166 (Thermo Fisher Scientific)	NM_001131020
*Hck* (299 bp, 58 °C)	Fw: TGA AGA AGG CAG CAA GCA G Rev: GCA GGA TAC CAA AGG ACC AG	PrimaQuant CYBR SL9902B (Steinbrenner Laborsysteme)	NM_001172117.1
*Aif1* (144 bp, 58 °C)	Fw: ATCAACAAGCAATTCCTCGATGA Rev: CAGCATTCGCTTCAAGGACATA	Absolute Blue qPCR SybrgreenMix AB-4166 (Thermo Fisher Scientific)	NM_019467
*Il1β* (348 bp, 55 °C)	Fw: GTGCTGTCGGACCCATATGAG Rev: CAGGAAGACAGGCTTGTGCTC Cy5-CAGCTG GAGAGTGTGGATCCCAAG C--PH FL-TAATGAAAGACGGCACACCCACCC	LightCycler 480 Probes Master 04887301001 (Roche)	NM_008361
*Il6* (471 bp, 55 °C)	Fw: TCGTGGAAA TGAGAAAAGAGTTG Rev: TATGCT TAGGCATAACGCACTAG Cy5--TGCTCTCCTAACAGATAAGCTGGAGTCAC--PH CATAAAATAGTCCTTCCTACCCCAATTTCC-FL	LightCycler 480 Probes Master 04887301001 (Roche)	NM_031168
*Lcn2* (239 bp, 58 °C)	Fw: TGGCCCTGAGTGTCATGTG Rev: CTCTTGTAGCTCATAGATGGTGC	Absolute Blue qPCR SybrgreenMix AB-4166 (Thermo Fisher Scientific)	NM_008491
*Mmp9* (106 bp, 58 °C)	Fw: AAGTCTCAGAAGGTGGAT Rev: AATAGGCTTTGTCTTGGTA	Absolute Blue qPCR SybrgreenMix AB-4166 (Thermo Fisher Scientific)	NM_013599
*Mpo* (220 bp, 58 °C)	Fw: ACACCCTCATCCAACCCTTC Rev: TGCTCAAATAGTCGCTCCC	PrimaQuant Cybr SL9902B (Steinbrenner Laborsysteme)	NM_010824.2
*Plat* (172 bp, 58 °C)	Fw: ACAACGACATCGCATTACTG Rev: TCAGAGAAGAATGGAGACGA	Absolute Blue qPCR SybrgreenMix AB-4166 (Thermo Fisher Scientific)	NM_008872
*Plau* (138 bp, 58 °C)	Fw: ACACTGCTTCATTCAACTCC Rev: CTGTCTTCCCTGTAGTATTCGT	Absolute Blue qPCR SybrgreenMix AB-4166 (Thermo Fisher Scientific)	NM_008873
*Ppia* (146 bp, 58 °C)	Fw: GCGTCTSCTTCGAGCTGTT Rev: RAAGTCACCACCCTGGCA	Absolute Blue qPCR SybrgreenMix AB-4166 (Thermo Fisher Scientific)	NM_008907
*Serpine1* (174 bp, 58 °C)	Fw: GGAYGTGARCTCATAGACA Rev: TGGTCGGAAAGACTTGTGA GCCTCCTCATCCTgCCTAAgTTCTCTC—FL LC Red640-GGAGACTGAAGTGGACCTCAGAGGGCC--PH	Maxima ProbeqPCR Master Mix K0262 (Thermo Fisher Scientific)	NM_008871
*Tnfα* (212 bp, 62 °C)	Fw: TCTCATCAGTTCTATGGCCC Rev: GGGAGTAGACAAGGTACAAC	Absolute Blue qPCR SybrgreenMix AB-4166 (Thermo Fisher Scientific)	NM_013693

Fw, forward; qPCR, real-time quantitative polymerase chain reaction; Rev, reverse.

### 2.7 Statistical analysis

All statistics were performed using GraphPad Prism 9. Data outliers identified by the ROUT test were removed, as indicated in the figure legends. Data distribution was tested by the Shapiro–Wilk test for each variable. For normally distributed data, comparisons were analyzed using the Student’s unpaired *t*-test; for variables not following a normal distribution, the Mann–Whitney U-test was used. Paired data without a normal distribution were analyzed by the Wilcoxon signed-rank test. Values from individual animals are shown as mean ± SD; *P* < .05 was considered statistically significant.

## 3 Results

### 3.1 PAR4^-/-^ does not affect TBI-induced neurological deficits

The CCI model of TBI induces acute neurological impairments that are particularly pronounced at 24 hours postinjury [39]. Mice were assessed for sensorimotor coordination using the RR performance test (Figure 1A) and for obvious neurological impairments using the NSS 1 day prior to and 1 day after TBI. PAR4^-/-^ mice showed an unexpected pretraumatic deficit in RR performance, as evidenced by a striking reduction in latency (PAR4^-/-^: 57.41 ± 18.93 seconds; BL6: 78.23 ± 20.01 seconds; *P* = .001, Student’s unpaired *t*-test; Figure 1C). This difference in RR performance was also observed at 24 hours after TBI (PAR4^-/-^: 29.00 ± 20.62 seconds; BL6: 46.25 ± 14.71 seconds; *P* = .0085, Student’s unpaired *t*-test; Figure 1C). Comparison of the pre- and posttraumatic values within the cohorts revealed no significant difference (Figure 1D).

The NSS, as a measure of neurological deficits, was almost zero before TBI and was greatly increased at 24 hours after TBI, as expected. However, the NSS was not different between PAR4^-/-^ and BL6 mice (Figure 1B).

### 3.2 PAR4^-/-^ causes increased mortality and body weight loss after TBI

**Figure 2:**
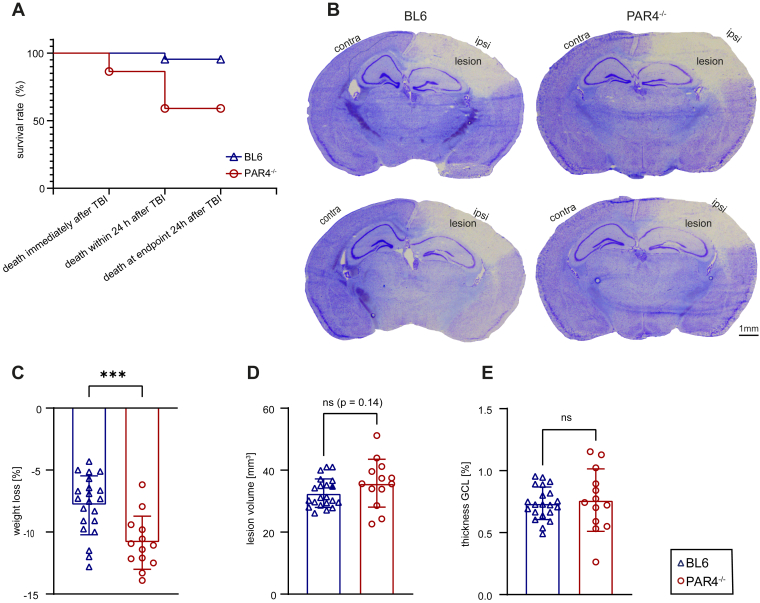
Mortality and loss of body weight were increased in PAR4-deficient (PAR4^-/-^) mice. PAR4^-/-^ did not affect lesion volume and gyrus granule cell layer (GCL) thickness. (A) Kaplan–Meier survival curve. (B) Cresyl violet-stained brain sections showing structural brain damage of PAR4^-/-^ and wild-type C57BL/6 (BL6) mice. (C) Loss of body weight 24 hours after traumatic brain injury (TBI). Values are expressed as a percentage relative to body weight before TBI. BL6: *n* = 21; PAR4^-/-^: *n* = 13; analysis by the Student’s unpaired *t*-test. (D) Lesion volume at 24 hours after TBI. Values are expressed as absolute lesion volume (mm^3^). BL6: *n* = 21; PAR4^-/-^: *n* = 13; analysis by the Student’s unpaired *t*-test. (E) Thickness of the GCL is given as the ratio of the ipsilesional (ipsi) hemisphere to the contralesional (contra) hemisphere. Values are expressed as a percentage relative to contra GCL thickness. BL6: *n* = 21; PAR4^-/-^: *n* = 13; analysis by the Student’s unpaired *t*-test. Values from individual animals and the mean ± SD are shown. ∗Indicates PAR4^-/-^ vs BL6; ∗*P* < .05; ∗∗*P* < .01; ∗∗∗*P* < .001; ∗∗∗∗*P* < .0001; ns, not significant.

The CCI model of TBI is associated with a low mortality rate, typically less than 10% in our laboratory. Consistently, 1 of 22 BL6 mice died within the 24-hour observation period after TBI, corresponding to a mortality rate of 4.5%. In contrast, 9 of 22 PAR4^-/-^ mice died during the same observation period, corresponding to a mortality rate of 40.9% (Figure 2A). The determination of body weight of the surviving mice following TBI revealed an increased body weight loss in PAR4^-/-^ mice compared with BL6 mice (PAR4^-/-^: −10.87% ± 2.144% vs BL6: −7.844% ± 2.379%; *P* = .0007, Student’s unpaired *t*-test; Figure 2C).

### 3.3 Structural brain damage is not different between PAR4^-/-^ and BL6 mice

The brain lesion volume was determined from cresyl violet-stained brain sections at 24 hours after TBI (Figure 2B). Only brains from mice that survived until 24 hours postinjury were included in this analysis. Despite the increased mortality rate of PAR4^-/-^ mice, brain lesion volumetry showed no differences between PAR4^-/-^ and BL6 mice (Figure 2D). Likewise, determination of the structural integrity of the hippocampal GCL (expressed as the ratio of ipsilesional to contralesional GCL thickness) showed a significant loss of neurons in the ipsilesional hemispheres but did not reveal differences between genotypes (Figure 2E).

### 3.4 PAR4^-/-^ impairs BBB integrity and aggravates intracerebral hemorrhage

**Figure 3:**
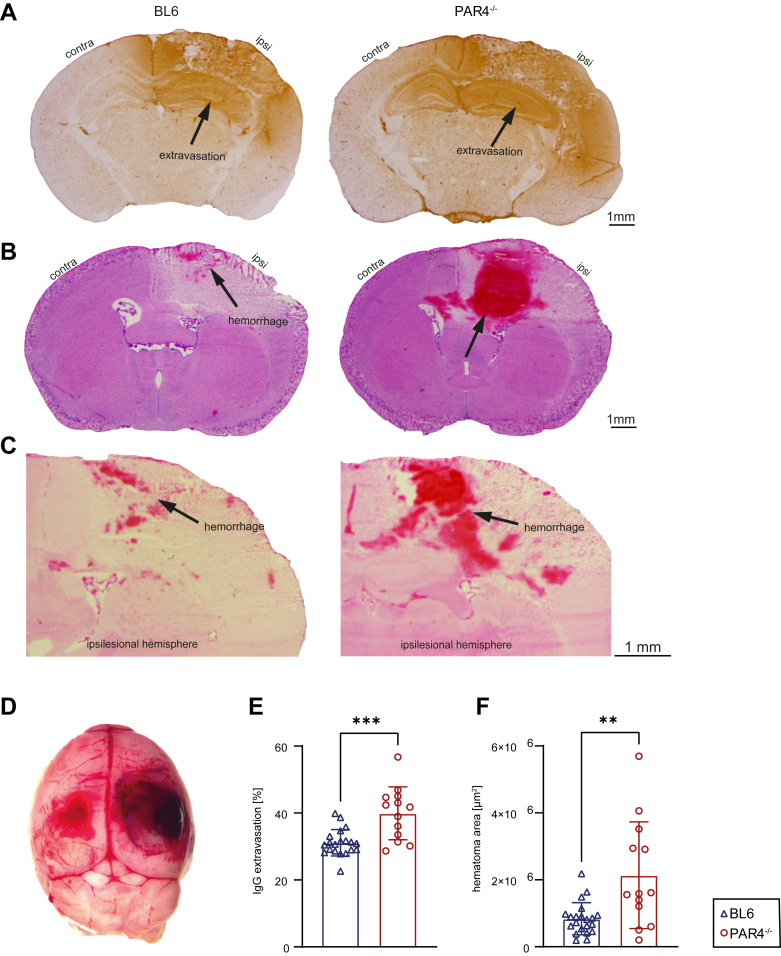
PAR4 deficiency (PAR4^-/-^) increases intracerebral hemorrhage and blood-brain barrier disruption. (A) Cryosections stained with immunoglobulin G (IgG) antibodies representing extravasation after traumatic brain injury. (B, C) Cryosections stained with hematoxylin and eosin showing intracerebral hemorrhage. (D) Exemplary picture taken after removal of the brain showing immense bleeding on the lesion side and in the contralesional (contra) subdural hematoma. (E) Area of IgG extravasation, expressed as a percentage of the total brain. One outlier was identified by the ROUT test and excluded. Wild-type C57BL/6 (BL6) mice: *n* = 20; PAR4^-/-^ mice: *n* = 13; analysis by the Student’s unpaired *t*-test. (F) Area of intracerebral hemorrhage given as absolute area (μm^2^). BL6: *n* = 21; PAR4^-/-^: *n* = 13; analysis by the Mann–Whitney U-test. Values from individual animals and the mean ± SD are shown. ∗Indicates PAR4^-/-^ vs BL6; ∗*P* < .05; ∗∗*P* < .01; ∗∗∗*P* < .001; ∗∗∗∗*P* < .0001; ns, not significant. Ipsi, ipsilesional.

We next tested whether PAR4^-/-^ affected IgG extravasation, resulting in loss of BBB integrity after TBI. We determined IgG extravasation using anti-IgG immunostaining (Figure 3A). Twenty-four hours after TBI, brain sections of PAR4^-/-^ mice unveiled a significantly enlarged IgG immunoreactive area in comparison with BL6 mice (PAR4^-/-^: 39.94% ± 7.903%; BL6: 31.68% ± 4.595%; *P* = .0005, Student’s unpaired *t*-test; Figure 3E).

Subsequently, we assessed whether increased IgG extravasation corresponds to augmented intracerebral hemorrhage. Intracerebral hemorrhage, illustrated by hematoxylin and eosin staining of the brain sections, demonstrated that the hematoma was significantly increased in PAR4^-/-^ mice (PAR4^-/-^: 2,137,786 ± 1,596,794 μm^2^; BL6: 838,113 ± 478,415 μm^2^; *P* = .008, Mann–Whitney U-test; Figure 3B, C, F). This finding was underlined by macroscopically detected subdural hemorrhage at the time of brain preparation (Figure 3D). Thus, PAR4^-/-^ affects BBB integrity and leads to extended hematoma, potentially causing a higher mortality rate.

### 3.5 The number of platelets and deposition are not different between PAR4^-/-^ and BL6 mice

**Figure 4:**
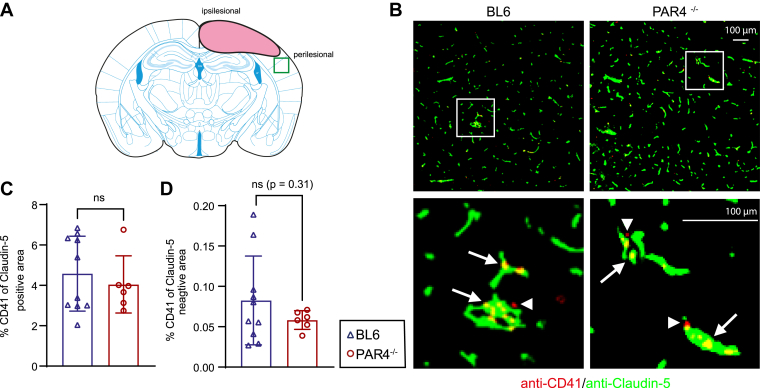
Platelet distribution is not different between PAR4-deficient (PAR4^-/-^) and wild-type C57BL/6 (BL6) mice. (A) Scheme showing the lesion core and perilesional region of interest for the examination of platelet distribution using CD41 and Claudin-5 double-immunostaining. (B) Images of anti-CD41/anti–Claudin-5 immunostaining. Boxed regions are shown at higher magnification; arrows and arrowheads point to CD41-positive platelets overlapping or nonoverlapping with Claudin-5, respectively. (C, D) CD41 percentage of Claudin-5–positive and Claudin-5–negative area as a proxy of intravasal and extravasal platelet distribution. BL6: *n* = 10; PAR4^-/-^: *n* = 6, analysis by the Student’s unpaired *t*-test. Values from individual animals and the mean ± SD are shown. ∗Indicates PAR4^-/-^ vs BL6; ∗*P* < .05; ∗∗*P* < .01; ∗∗∗*P* < .001; ∗∗∗∗*P* < .0001; ns, not significant.

To test the possibility that inherent alterations in platelet aggregation may contribute to increased intracerebral hemorrhage in PAR4^-/-^ mice, we examined CD41/Claudin-5 double-immunostaining in the perilesional cortex at 24 hours after TBI (Figure 4A, B). We characterized CD41-positive platelets by their spatial relationship to the endothelial blood vessel marker Claudin-5 as a proxy for intravascular and extravascular platelets in a subset of animals (Figure 4C, D). However, this analysis did not reveal differences in platelet aggregation between genotypes (%CD41 of Claudin-5–positive area: PAR4^-/-^: 4.06% ± 1.416% vs BL6: 4.580% ± 1.856%; *P* = .555, Student’s unpaired *t*-test; Figure 4C; %CD41 of Claudin-5–negative area: PAR4^-/-^: 0.058% ± 0.012% vs BL6: 0.083% ± 0.055%; *P* = .31, Student’s unpaired *t*-test; Figure 4D).

### 3.6 Gene expression is hardly affected by PAR4^-/-^

**Figure 5:**
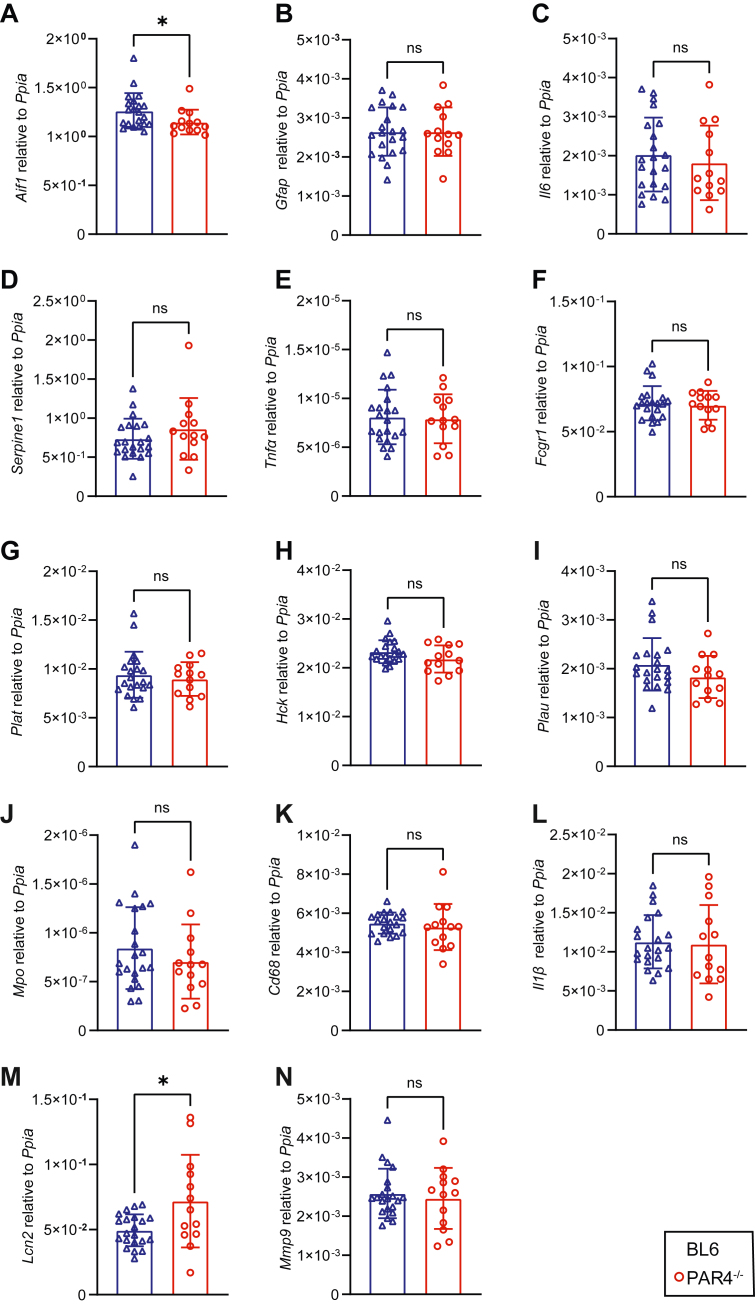
PAR4 deficiency (PAR4^-/-^) increases expression of Lcn2 and decreases expression of Iba1. (A–N) Real-time quantitative polymerase chain reaction (results for mRNA in the ipsilesional hemisphere). Results are presented relative to the housekeeping gene Ppia. (K) One outlier was identified by the ROUT test and excluded. Wild-type C57BL/6 (BL6) mice: *n* = 20; PAR4^-/-^ mice: *n* = 13. (A–J, L–N) BL6: *n* = 21; PAR4^-/-^: *n* = 13. (A–N) Analysis by the Mann–Whitney U-test or Student’s unpaired *t*-test. Values from individual animals and the mean ± SD are shown. ∗Indicates PAR4^-/-^ vs BL6; ∗*P* < .05; ∗∗*P* < .01; ∗∗∗*P* < .001; ∗∗∗∗*P* < .0001; ns, not significant.

Substantial screening of several proinflammatory cytokines (interleukin-1β, interleukin-6, and tumor necrosis factor-α), a microglial activation marker (Iba1), an astrocyte marker (glial fibrillary acid protein), a monocyte/macrophage marker (CD68), a neutrophil marker (myeloperoxidase), IgG activity (Fcgr1 and Hck), a marker of extracellular matrix breakdown (matrix metalloproteinase-9), an acute-phase protein released after brain injury by reactive astrocytes (Lcn2), and coagulation-related proteins (tissue-type plasminogen activator (tPA), urokinase-type plasminogen activator, and Serpine1) was performed, all of which were mainly upregulated in the early phase after TBI (Figure 5). Iba1 expression levels were significantly decreased in PAR4^-/-^ mice at 24 hours post-TBI (PAR4^-/-^: 1.147 ± 0.1267; BL6: 1.263 ± 0.1807; *P* = .027, Mann–Whitney U-test; Figure 5A), indicating reduced microglial activity. In contrast, expression levels of Lcn2 were significantly increased in PAR4^-/-^ mice (PAR4^-/-^: 0.07190 ± 0.03560; BL6: 0.04942 ± 0.01226; *P* = .0118, Student’s unpaired *t*-test; Figure 5M). Upregulation of Lcn2 suggests increased secretion by especially activated astrocytes, consequently promoting neuroinflammation.

## 4 Discussion

In this study, we examined the acute effects of PAR4^-/-^ in mice following TBI. Our results show a crucial role of PAR4 for cerebral hemostasis. PAR4^-/-^ mice exhibited higher mortality, impaired hemostasis, and exacerbated BBB impairment, without a difference in TBI-induced neurological deficits between groups.

Contrary to our results in the CCI model of TBI, PAR4^-/-^ mice showed no differences in survival rates and attenuated brain tissue injury following ischemic stroke in the tMCAO model [24]. The use of PAR4 antagonist ML354 in tMCAO reduced brain damage and improved neurological outcome [25]. This discrepancy is likely due to differences in bleeding risk. Although hemorrhagic transformation due to reperfusion injury or BBB disruption can occur in the MCAO stroke model, it occurs mainly after treatment with tPA [41,42]. In contrast, in the CCI model of TBI, bleeding can occur because of physical trauma to blood vessels, and immediate bleeding is common, with a risk of delayed bleeding in the following hours to days [[43], [44], [45]]. Similar considerations also apply to clinical scenarios of ischemic stroke and TBI [[46], [47], [48], [49]]. However, there is a lack of studies using PAR4^-/-^ mice following experimental TBI.

Given thrombin's crucial role in secondary brain injury, researchers are focusing on PAR4 antagonism as a potential therapeutic approach [50]. The selective PAR4 antagonist BMS-986120 revealed reduced thrombin-induced inflammation in astrocytes after TBI [29]. Notably, the previously described neuroprotective effects of ML354 appear to be achieved through the inhibition of neuronal PAR4 rather than its platelet counterpart [25]. Nevertheless, this aspect of platelet count has garnered attention as the PAR1 antagonist vorapaxar demonstrates an increased risk of bleeding in patients with myocardial infarction [17]. In contrast, targeting PAR4 as a therapeutic strategy appears to offer effective antithrombotic benefits with fewer side effects, particularly in patients with cardiovascular conditions [18].

In this study, we found that PAR4^-/-^ mice exhibited severe impairment of cerebral hemostasis after CCI. Interestingly, newborn PAR4^-/-^ pups exhibited spontaneous bleeding in various organs as well as perinatal lethality [51]. These findings are consistent with previous studies reporting prolonged bleeding times in PAR4^-/-^ mice due to impaired secondary hemostasis [21,22,52,53]. Mouse mutants deficient in other coagulation factors, such as factor XI, plasminogen activator inhibitor-1, urokinase-type plasminogen activator, and tPA, do not exhibit such a severe phenotype after TBI [7,54,55]. These models provide valuable insights into the mechanisms of blood clotting and wound healing in the brain, enabling the study of the complex interplay among coagulation factors, platelets, and vascular integrity in the context of brain injury and hemostasis. The PAR4 model that imitates maximal pharmacological inhibition may provide several advantages, including a comprehensive understanding of platelet reactivity and thrombotic processes, as well as insights into neuroinflammatory mechanisms. Numerous studies investigating PAR4 antibodies, PAR4 point mutations, or PAR4 antagonists (in patients with coronary artery disease) have shown antithrombotic effects [[56], [57], [58], [59], [60], [61]]. Unfortunately, an effective antiplatelet agent with minimal side effects for patients with a history of stroke, myocardial infarction, transient ischemic attack, or intracerebral hemorrhage is still unavailable. However, several lines of research aim to comprehend the underlying mechanisms to develop suitable PAR4 antagonists for therapeutic use [56,[62], [63], [64], [65]]. Nonetheless, the transferability of the PAR4 investigation results from mice should be approached with caution, as PAR4 signaling in human platelets differs from that in mouse platelets [66,67]. In mice, platelets are characterized by the presence of PAR3 and PAR4, while in humans, PAR1 and PAR4 are the main thrombin receptors on platelets. In mouse platelets, PAR3 acts as a cofactor for PAR4 and lacks independent signaling capability. Conversely, human PAR1 can be triggered by low levels of thrombin, initiating an intracellular signaling cascade without the involvement of PAR4. Thus, human platelets can still respond to thrombin via PAR1 even when a PAR4 antagonist is present, whereas PAR4^-/-^ mice do not respond to thrombin [16,67,68].

Previously mentioned research has established that PAR4 plays a crucial role in both neuroinflammation and hemostasis. Additionally, it is widely recognized that TBI results in a complex interplay between coagulation and inflammatory pathways. After TBI, the release of TF leads to thrombin generation [9]. The activation of PAR4 by high levels of thrombin can exacerbate brain damage through inflammatory mechanisms [29]. TF has diverse functions in hemostasis, thrombosis, and inflammation, and various studies have pinpointed the TF-mediated pathway of thrombin generation after TBI, both in coagulation activation after injury and in traumatic coagulopathy [9,14,69]. As thrombin is a downstream factor of TF in the extrinsic pathway, a reciprocal pattern of interaction might exist between TF, thrombin, and PAR4. However, the precise pathomechanisms still need to be clarified. For example, systemic hypercoagulability in a cohort of TBI patients, characterized by increased clot strength and decreased fibrinolysis, occurred independently of TF levels [70].

IgG extravasation is generally considered a valid parameter to assess BBB impairment. However, its reliability may be compromised when increased bleeding is observed. Bleeding allows blood components, including IgG, to enter the brain tissue directly. This may lead to false-positive results because the presence of IgG may not reflect only BBB permeability. Distinguishing between primary BBB disruption and the secondary effects of bleeding becomes difficult. Combining IgG extravasation data with other markers of BBB integrity, for example, measuring tight junction proteins (eg, Claudin-5 and occludin), may be a possible strategy to directly assess BBB integrity. While IgG extravasation can provide valuable information about BBB compromise, its interpretation requires caution and consideration of potential confounding factors when increased bleeding is present. The use of multiple complementary methods may allow for a more comprehensive assessment of BBB integrity in such situations. As PAR4^-/-^ impaired BBB integrity and aggravated intracerebral hemorrhage in our CCI model, one would assume an increase in brain lesion volume, which was not detected. An explanation may involve the delicate balance of cerebral hemostasis, which, on the one hand, aggravates TBI by forming clots due to decreased intracerebral blood flow and, on the other hand, increases intracerebral bleeding due to a lack of clots and vascular damage. However, our immunostaining of brain cryosections did not provide evidence for alterations in clot formation in the perilesional cortex between genotypes. Moreover, we cannot exclude the possibility that this result was due to limited validity regarding the intra- and extravascular deposition of platelets, which could be addressed in future studies, eg, by *in vivo* imaging techniques.

The inflammatory cascade after TBI is complex and dynamic. Peak expression of different inflammatory markers may occur at varying time points, and 24 hours may not capture the full extent of the inflammatory response. A single 24-hour time point may not capture the dynamic changes in inflammatory marker expression, which could fluctuate rapidly or show delayed responses.

Unfortunately, due to the increased vulnerability of PAR4^-/-^ mice, which resulted in poor survival outcomes and associated ethical considerations, it was not possible to study longer survival time points.

Prior to this study, it was generally believed that PAR4^-/-^ mice were phenotypically normal, appearing similar in size and overall appearance to BL6 mice [52]. In this research, even before subjecting the mice to TBI, the PAR4^-/-^ group demonstrated poor performance on the rotating rod test, a standard assessment of motor coordination and balance. Earlier work revealed the presence of PAR4 in pyramidal neurons in layers 3 and 5 of the neocortex, which are critical for motor control [71]. Additionally, PAR4 was found to be expressed in brain regions responsible for sensory processing and motor function [25]. Furthermore, *in situ* hybridization data for F2RL3 mRNA suggest cerebellar expression of PAR4 [72]. This distribution pattern of PAR4 in the brain provides a potential explanation for the observed motor deficits in PAR4^-/-^ mice, even in the absence of injury. .However, as no littermate controls were given, which is a limitation of the study, genetic divergence cannot be excluded. Another limitation of the study is potential survival bias, as the experiment was stopped after 24 hours, followed by the quantification of the lesion only in mice that survived the observation period. Therefore, we cannot rule out that mice that died before the 24-hour endpoint as a result of TBI, potentially exhibiting increased intracerebral hemorrhage, may have shown larger lesions and heightened inflammatory responses to TBI.

In conclusion, this study highlights the crucial role of PAR4 following TBI. PAR4^-/-^ resulted in devastating consequences, and PAR4^-/-^ mice may serve as a model for future studies examining pharmacological compounds to optimize hemostasis management following TBI.
